# Supplementary figures and images for: Metabolite Profile of Treatment-Naive Metabolic Syndrome Subjects in Relation to Cardiovascular Disease Risk
Source: Metabolites. 2021 Apr 13;11(4):236. doi: 10.3390/metabo11040236 (PMC8069178; doi:10.3390/metabo11040236)

Receiver operating characteristic

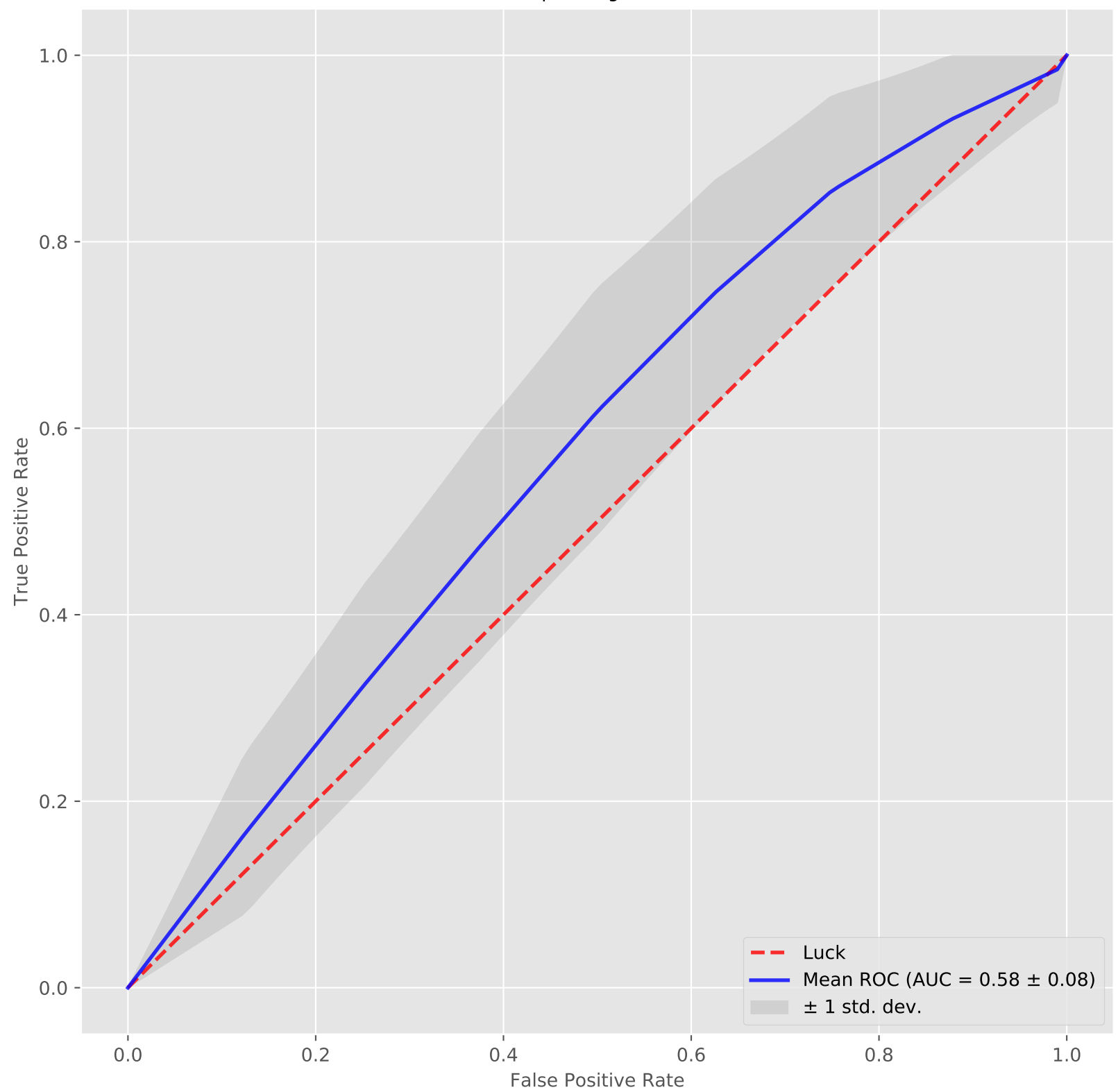

Supplement: Supplementary file 1 [file metabolites-11-00236-s001.zip › FigureS1 AUC_ML_Rd_IR_varia_select.pdf]

Receiver operating characteristic

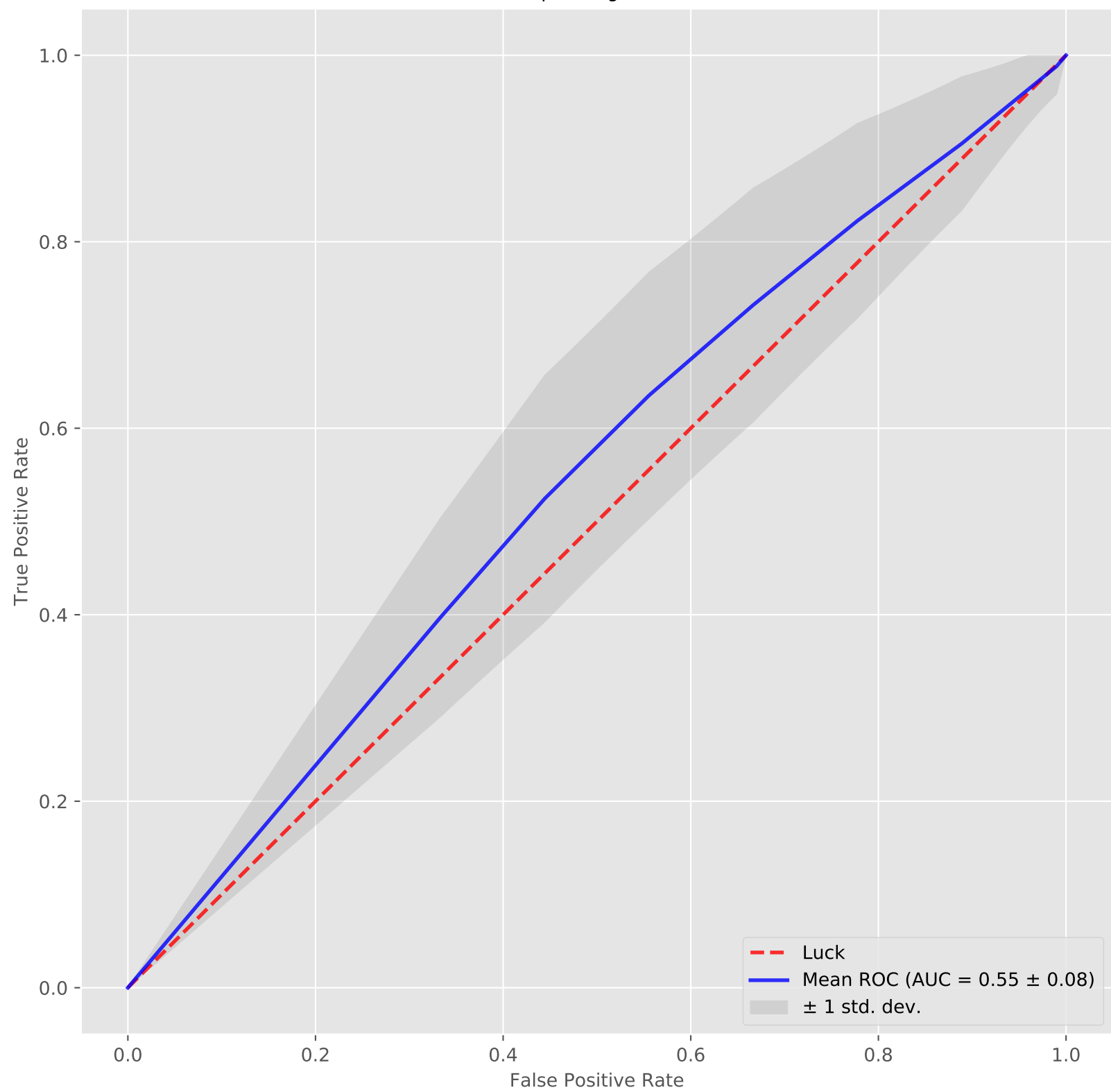

Supplement: Supplementary file 1 [file metabolites-11-00236-s001.zip › FigureS2 Plot_AUC_top9metabolites.pdf]
